# Adjuvant Treatment of Stage I–II Serous Endometrial Cancer: A Single Institution 20-Year Experience

**DOI:** 10.3390/curroncol31070277

**Published:** 2024-06-29

**Authors:** Aquila Akingbade, François Fabi, Rodrigo Cartes, James Tsui, Joanne Alfieri

**Affiliations:** 1 Division of Radiation Oncology, London Health Sciences Center, Western University, London, ON N6A 5W9, Canada; aquila.akingbade@lhsc.on.ca; 2 Radiation Oncology Service, Centre Intégré de Cancérologie (CIC), Hôpital de l’Enfant-Jésus, Centre Hospitalier Universitaire de Québec, Québec, QC G1J 1Z4, Canada; francois.fabi.1@ulaval.ca; 3 Division of Radiation Oncology, McGill University Health Center, Montreal, QC H4A 3J1, Canada; rodrigo.cartes.indo.med@ssss.gouv.qc.ca (R.C.); james.tsui.med@mcgill.ca (J.T.)

**Keywords:** early-stage, serous endometrial cancer, adjuvant therapy

## Abstract

*Background:* Serous endometrial carcinoma (SEC) is a high-risk subtype of endometrial cancer. The effectiveness of multiple adjuvant therapies, namely chemotherapy (CT), radiotherapy (RT), and sequential/concurrent chemotherapy with radiotherapy (CRT), have previously been investigated. However, optimal management of early-stage SEC remains unclarified. *Methods:* All cases of early-stage SEC (FIGO 2009 stages I–II) treated in our institution from 2002 to 2019 were identified. Patient data were documented until September 2023. Overall survival (OS) and disease-free survival (DFS) were computed using Kaplan–Meier estimates and Cox’s proportional hazard model; descriptive statistical analysis was performed. *Results:* A total of 50 patients underwent total hysterectomy-bilateral salpingo-oophorectomy and omentectomy, displaying stage IA (60%), IB (24%), and II (16%) disease. The median follow-up was 90.9 months. Patients underwent adjuvant CRT (*n* = 36, 72%), CT (*n* = 6, 12%), or RT (*n* = 6, 12%). Two patients were observed and excluded from analyses. The 42 patients who received radiotherapy had pelvic external beam radiotherapy (*n* = 10), vaginal brachytherapy (*n* = 21), or both (*n* = 11). CRT had better OS (HR 0.14, 95%CI 0.04–0.52, *p* < 0.005) and DFS (HR 0.25, 95%CI 0.07–0.97, *p* = 0.05) than CT alone. RT displayed no OS or DFS benefits compared to CT/CRT. Recurrences were mostly distant. Acute and late G3-4 toxicities were primarily hematologic. *Conclusions:* Our data underline the challenge of treating SEC. CRT appears to be superior to CT alone but not to RT. Most recurrences were distant, highlighting the need for optimized systemic treatment options.

## 1. Introduction

Endometrial cancer (EC) is the fourth most prevalent malignancy among Canadian women, with 8000 new diagnoses and 1400 deaths in 2021 [1]. Despite 75–80% of newly diagnosed cases being classified as FIGO stage I, there is a concerning upward trend in EC-specific mortality [1,2]. EC is categorized histologically into type I, endometrioid EC, and type II, non-endometrioid EC, which includes serous endometrial carcinoma (SEC) and clear cell carcinoma (CCC), among other subtypes. Though constituting only 10% of all EC diagnoses, SEC exhibits aggressive clinical behavior, contributing to about 40% of EC-related deaths [2]. Women with CCC and SEC typically present with more advanced clinicopathologic stages and experience poorer outcomes compared to those with endometrioid histology [3,4]. Notably, the 5-year disease-specific survival rate differs significantly when comparing SEC and CCC with high-grade endometrioid EC (respectively, 55% and 68% vs. 77%, *p* < 0.0001). This distinction persists even after adjusting for stage, with rates of 74%, 82%, and 86% (*p* < 0.0001) observed for the combined analysis of stage I and II of SEC, CCC, and high-grade endometrioid EC, respectively [5]. Consequently, non-endometrioid histologies with myometrial invasion are classified as high-risk in current clinical guidelines, while those without myometrial invasion are deemed intermediate-risk [6]. Although not yet incorporated into clinical guidelines, the latest FIGO 2023 staging system considers SEC an aggressive histology and upgrades tumors without myometrial invasion to stage IC and those with any extent of myometrial invasion to stage IIC [7].

Primary treatment for SEC patients eligible for surgery involves total hysterectomy and bilateral salpingo-oophorectomy (TH-BSO), accompanied by comprehensive surgical staging with pelvic lymph node dissection or sentinel node biopsy, omentectomy, and peritoneal washings [8,9,10,11]. Given SEC’s heightened risk of recurrence and decreased overall survival (OS), careful consideration of adjuvant treatment is essential. However, due to its rarity, patients with SEC are often underrepresented in clinical trials, leading to decision-making primarily relying on subgroup analyses and retrospective studies. These uncertainties are further compounded by discrepancies regarding the potential survival benefits of various adjuvant strategies in this patient subgroup [12,13]. Consequently, there remains no consensus on the optimal treatment approach for early-stage SEC (FIGO 2009 stage IA, IB, and II). A range of options exists, including observation, radiotherapy (RT) in the form of pelvic external beam radiotherapy (pEBRT) or vaginal vault brachytherapy (VBT), chemotherapy (CT), or combined radiotherapy and chemotherapy (CRT), administered either sequentially and/or concurrently [6,13,14,15,16,17].

This retrospective study aims to enhance the SEC literature by evaluating the impact of various adjuvant treatment modalities in patients with stage I–II SEC on disease-free survival (DFS), overall survival (OS), treatment-related acute and late toxicity, and recurrence patterns.

## 2. Materials and Methods

### 2.1. Design and Participants

After IRB approval, we retrospectively identified all SEC cases treated at McGill University Health Center (MUHC) between January 2002 and December 2019. Eligible patients had histologically confirmed SEC and underwent complete surgical staging, including TH-BSO, pelvic nodal staging via either pelvic lymphadenectomy or sentinel lymph node biopsy (SLNB), omentectomy, and peritoneal washings. Patients with SEC and FIGO 2009 stages IA (endometrium-confined or <50% invasion of the myometrium), IB (≥50% myometrial invasion, MI), and II (cervical stroma invasion) were included, with documentation of MI status for stage IA cases. Patients with stage IA without MI were classified as intermediate-risk, and the remaining as high-risk considering current clinical guidelines, despite the upgrade aggressive histologies had in the 2023 FIGO staging system [7]. Patients with FIGO 2009 stage III SEC (serosal, adnexal, vaginal, or parametrial involvement), positive lymph nodes, positive peritoneal washing, or stage IVA/B (bladder/bowel mucosa involvement or distant disease) or non-SEC histologies were excluded.

### 2.2. Data Collection

Data were extracted from written and electronic records, including age at diagnosis, surgery date, surgery type, surgical stage, lymphovascular invasion (LVI) extent according to the pathologist review, adjuvant treatment duration, recurrence details, and survival status. Post-operative management was documented as observation, RT (pEBRT and/or VBT), CT, or CRT (sequential or concurrent). Treatment specifications were recorded. pEBRT entailed delivering a total dose of 45 Gray (Gy) over 25 daily fractions, administered five times per week. Target areas included the surgical bed, upper vagina, external, internal, and distal common iliac lymph nodes, as well as pre-sacral nodes for stage II, utilizing either four-field 3D-conformal radiotherapy (3D-CRT) or intensity-modulated radiation therapy (IMRT). Since 2011, the local policy for pEBRT has included IMRT exclusively. Vaginal vault brachytherapy (VBT) was administered to the upper third of the vagina either as monotherapy with a total dose of 21 Gy over 3 weekly fractions or as a single-fraction 6 Gy boost. Chemotherapy (CT) predominantly comprised carboplatin (AUC 6) combined with paclitaxel (175 mg/m^2^ over three hours) administered every three weeks for six cycles. Single-agent carboplatin (AUC 5–6) was considered when patients were unable to tolerate the combination. Chemoradiotherapy (CRT) involved the sequential or concurrent administration of pEBRT and/or VBT using the aforementioned chemotherapy regimens. Post-treatment, patients had regular clinical examinations with imaging/biopsies as indicated.

### 2.3. Outcomes

The main outcome was OS, defined as the duration of patient survival from the time of adjuvant treatment initiation until death from any cause. Secondary outcomes included DFS, patterns of failure, and acute and late toxicity. DFS was measured from the moment of treatment initiation until the diagnosis of recurrence by imaging or pathology. Failure was classified as loco-regional (vagina or pelvis), distant (outside the true pelvis), or combined if it included a combination of the former. Treatment-related genitourinary (GU), gastrointestinal (GI), and hematologic toxicity was recorded and graded per the National Cancer Institute Common Terminology Criteria for Adverse Events version 4.0 (CTCAE v4.0). Toxicity was classified as acute with presentation during adjuvant oncologic treatment and up to 12 weeks from the end of adjuvant treatment or late if it was reported beyond this period.

OS data were collected up to the censoring date. DFS, patterns of failure, and acute and late toxicity data were censored at the last follow-up visit, positive staging imaging or pathology. Loss to follow-up was defined as last seen >2 years prior to the censoring date.

### 2.4. Statistical Analysis

Patient characteristics, adjuvant treatment, pathology, acute and late toxicity, and recurrence patterns were reported with descriptive statistics. Kaplan–Meier estimates were utilized to compute OS and DFS. Log-rank and Cox’s proportional hazard model were used to analyze differences between treatments. All calculated *p*-values were two-sided, with values < 0.05 considered statistically significant. Analyses were conducted using the lifelines library version 0.27.8 in Python v3.10.12.

## 3. Results

### 3.1. Patient and Treatment Characteristics

Between 2002 and 2019, 50 patients with stage I–II SEC received treatment at our center. Population baseline characteristics are outlined in Table 1. The median age at diagnosis was 69 years. FIGO 2009 stage IA was the most common (60%), followed by stage IB (24%) and stage II (16%). Most stage IA tumors were confined to the endometrium (67%). LVI was negative in 46% of patients, focal in 12%, and extensive in 42%. Notably, when known (*n* = 42), most tumors exhibited p53 mutation (*n* = 40, 95%). p53 status was wild-type in 2 patients and unknown in 8 patients (16%). Post-surgery management included CT for 6 patients (12%), RT for 6 patients (12%), CRT for 36 patients (72%), and observation for 2 patients (4%). Patients undergoing observation were excluded from survival and toxicity analyses, as well as patterns of recurrence assessment.

Radiotherapy was administered as VBT alone (21 Gy in 3 fractions), pEBRT alone (45 Gy in 25 fractions), or a combination of both (pEBRT, 45 Gy in 25 fractions plus VBT boost, 6 Gy in 1 fraction). In the RT group, half received pEBRT (*n* = 3, 50%), while in the CRT group, VBT alone (*n* = 19, 53%) was the most frequently employed approach. pEBRT plus VBT boost was utilized in *n* = 10 (28%) of the CRT group and *n* = 1 (17%) of the RT group, more frequently in patients with extensive LVI and/or stage II disease. Most patients receiving pEBRT were treated with IMRT (83%), while the remainder, all treated before 2011, underwent 3D-CRT.

Carboplatin-paclitaxel combination for 4 or 6 cycles (*n* = 11 and *n* = 23, respectively) was the most commonly prescribed chemotherapy regimen in both the CT (*n* = 5, 83%) and CRT (*n* = 29, 81%) groups, accounting for 34 patients. Carboplatin administered as a single agent for 4 or 6 cycles (*n* = 5 and *n* = 2, respectively) was the second most frequently prescribed regimen, accounting for 17% in each group (CT; *n* = 1 and CRT; *n* = 6).

The CRT group predominantly underwent sequential chemoradiation (*n* = 29, 81%). Concomitant CRT was administered to 7 patients, with 5 receiving VBT alone (in between 2 cycles of chemotherapy), 1 receiving a VBT boost alongside carboplatin-paclitaxel chemotherapy followed by pEBRT, and 1 patient enrolled in the RTOG 0921 trial receiving concomitant pEBRT with cisplatin and bevacizumab, followed by adjuvant carboplatin-paclitaxel.

### 3.2. Survival Outcomes

**Figure 1:**
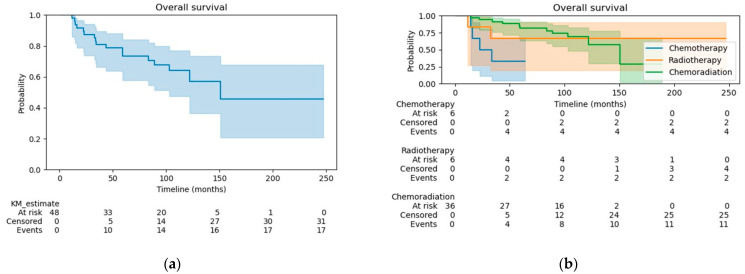
(**a**) Overall survival for the whole cohort; (**b**) Overall survival by treatment group.

**Figure 2:**
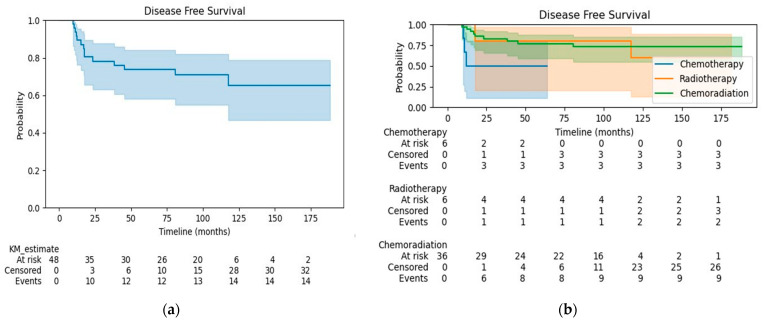
(**a**) Disease-free survival for the whole cohort; (**b**) Disease-free survival by treatment group.

The median follow-up was 90.9 months for all patients, 28.3 months for the CT group, 141.9 months for the RT group, and 97.6 months for the CRT group. At the time of analysis, 17 (35.4%) patients had died. The OS at 3 years was 81% for all patients (Figure 1a), 33% for the CT group, 67% for the RT group, and 91% for the CRT group (Figure 1b). OS was significantly better in patients treated with CRT compared to CT alone (HR 0.14, 95% CI 0.04–0.52, *p* < 0.005). There were no differences in OS when comparing patients in the RT alone group with patients receiving CRT or CT alone (log-rank test *p* = 0.72 and 0.32, respectively). The disease-free survival (DFS) at 3 years was 78% for all patients (Figure 2a), 50% for the CT group, 80% for the RT group, and 83% for the CRT group (Figure 2b). Patients treated with CRT had significantly better DFS than those in the CT alone group (log-rank *p* = 0.03; HR 0.25, 95% CI 0.07–0.97, *p* = 0.05). The DFS did not differ significantly between the CRT and RT alone groups (log-rank test *p* = 0.76) or between patients treated with RT alone vs. CT alone (log-rank test *p* = 0.18).

### 3.3. Patterns of Recurrence

Recurrence occurred in 14 patients (29%); 11 of them (79%) have died (Table 2). The most common pattern was distant failure in 9 patients (64%), with similar rates observed between the CRT and CT groups. The second most common pattern was combined loco-regional and distant recurrence, observed in 3 patients (21.4%), with the highest rate seen in the RT alone group. In these patients, the loco-regional component of recurrence was either in regional nodes in patients treated with VBT or at the field border in those who received pEBRT. There were no in-field recurrences. The sites of metastases at the time of recurrence included the lungs (5 patients), distant lymph nodes (5 patients), liver (2 patients), peritoneum (3 patients), and brain (1 patient). Loco-regional failure alone was observed in only 2 patients, both in the CT group (Table 3).

Only 3 of the 14 patients (21%) who experienced recurrence are still alive: one patient presenting with lower vaginal failure and inguinal node metastases (RT group), treated with salvage RT (45 Gy in 25 fractions) and currently free of disease for 9.3 years; one patient with lung metastases (CRT group) treated with stereotactic body radiation therapy (SBRT) twice (48 Gy in 3 fractions and 30 Gy in 1 fraction) and currently showing a possible third out-of-field lung progression; and one patient presenting with lung metastases treated with chemotherapy and subsequent lines of systemic therapy, currently experiencing progressive disease for the 4th time with bilateral pleural effusion and stable lung nodules. See supplemental document for details of re-irradiation of 4 patients who previously received radiotherapy as part of their initial adjuvant treatment.

When assessing by risk, the failure rate in patients with intermediate-risk disease was 25% (*n* = 4). All four patients were in the CRT group receiving predominantly VBT as RT (75%). Distant failure alone was observed in 3 patients, while one patient who received VBT presented with regional and non-regional adenopathy. High-risk patients had a recurrence rate of 35.7% (*n* = 10), with CRT being the most common adjuvant modality in 50% of these patients, mainly in the form of pelvic RT with or without VBT boost (80%). The majority of high-risk patients had non-regional nodes or distant metastases (80%) as a component of their recurrence. However, two patients (20%) treated with CT alone presented with isolated loco-regional failure.

### 3.4. Toxicity Outcomes and Treatment Compliance

Table 4 summarizes the patterns of acute and late toxicity. Acute GU toxicity was uncommon, as most patients reported no urinary symptoms (85%, *n* = 41). Acute GI toxicity was frequent but generally mild, with 73% of patients (*n* = 35) experiencing G1-G2 events and 25% (*n* = 12) without any GI symptoms. Acute hematological toxicity was also frequent, with most patients presenting with G1-G2 events (*n* = 27) or no toxicity (*n* = 10), although there was a higher incidence of severe events.

Overall, 13 of the 48 patients (27%) experienced acute G3+ events. There were 5 events in the CT group (4 of 6 patients, 67%), 10 events in the CRT group (9 of 36 patients, 25%), and none in the RT alone group, with two patients experiencing more than one G3+ event. The most common acute G3+ toxicity was hematological, observed in 12 patients (25%): 4 in the CT group and 8 in the CRT group while receiving chemotherapy. Among these patients, 5 had G4 neutropenia leading to febrile neutropenia or sepsis that required hospitalization. The second most common acute G3+ toxicity was GI, observed in 2 patients in the CRT group who required IV hydration due to vomiting while receiving chemotherapy. There were no G4 acute GI events. Acute G3+ GU toxicity was observed in only one patient, who presented with a G4 acute fistula in the CT alone group.

Severe chronic toxicity was infrequent in all groups and observed in only 3 patients. Despite frequent acute G3+ hematological toxicity, G3+ late events were reported in only 2 patients (4%), both in the CRT group. G3+ GU chronic toxicity was observed in only one patient (2%) in the CT group, who developed a chronic GU fistula. There were no cases of G3+ late GI events. The highest chronic GI toxicity observed was G1 in 6 patients and G2 in 2 patients (*n* = 1 in the CT group; *n* = 2 in the RT group; *n* = 3 in the CRT group).

Treatment compliance by treatment group was 67% for the CT group, 100% for the RT group, and 69% for the CRT group. Among all patients who received chemotherapy, 29 of 42 patients (69%) completed all initially planned cycles. By regimen, all patients receiving single-agent carboplatin completed their chemotherapy as initially planned (*n* = 1 in the CT group and *n* = 6 in the CRT group), while only 62% (*n* = 21) completed the originally planned carboplatin-paclitaxel cycles. By treatment group, 67% (*n* = 4) of the patients in the CT group and 64% (*n* = 23) of the patients in the CRT group completed all cycles of chemotherapy as initially planned. Among patients receiving RT, the total dose and number of fractions of pEBRT and/or VBT were completed by 100% (*n* = 42) of the patients in both the RT group (*n* = 6) and the CRT group (*n* = 36). 

When analyzing compliance by toxicity, among the 13 patients experiencing G3+ acute toxicity, only 54% (7 patients) were able to complete the planned number of chemotherapy cycles (2 of 4, 50%, in the CT group and 5 of 9, 55%, in the CRT group). Among the patients experiencing severe acute events, all those who had radiotherapy in their treatment plan were able to complete their RT as planned (*n* = 4 pEBRT, *n* = 3 VBT, *n* = 2 pEBRT + VBT boost).

## 4. Discussion

Our retrospective study exclusively examined women with early-stage SEC. Most patients had their p53 status assessed, with more than 95% having p53 mutated tumors, aligning with reports from The Cancer Genome Atlas (TCGA) showing a prevalence of 90% TP53 mutations in SEC [18]. Pathological assessment included only FIGO 2009 stage I–II tumors, with 60% being stage IA. Among these patients, two-thirds presented with no myometrial invasion. Therefore, our outcomes reflect the characteristics of a cohort comprising intermediate (40%) and high-risk (60%) populations according to current clinical guidelines [5,6].

Overall, the most frequent treatment modality was a combination of chemotherapy with VBT alone, commonly administered sequentially. In the CRT group, patients eligible for VBT alone tended to present with stage IA, without LVI. In contrast, those with stage IB or II with focal or extensive LVI were typically treated with pEBRT +/– VBT boost. 

Two large population-based studies and a large multi-institutional retrospective cohort have suggested a survival benefit of adjuvant chemotherapy compared to observation in patients with early-stage SEC, even without myometrial invasion [18,19,20]. This potential benefit was also observed in patients receiving VBT alone or in combination with CT compared to observation in early-stage SEC [20]. However, this population of patients was entirely excluded or underrepresented in large randomized phase III trials exploring the role of adjuvant VBT or WPRT alone, such as GOG-99, PORTEC-1, and PORTEC-2 [21,22,23]. In contrast, PORTEC-3 demonstrated survival benefits in patients with high-risk disease with the combination of chemotherapy and pelvic radiotherapy compared to pelvic RT alone, and this benefit persisted in the subgroup analysis for serous histology [13]. Subsequent phase III trials like GOG-249 and GOG-258 included a proportion of stage I–II SEC (14.6% and 2.2%, respectively) but did not find survival benefits with CRT compared to RT alone or CT alone, respectively. However, CRT in GOG-249 was administered in the form of VBT, and patients in GOG-258 mainly presented with locally advanced endometrioid disease [13,24,25].

For patients with stage IA SEC without myometrial invasion, observation or VBT alone or in combination with systemic therapy are treatment options [6,14]. A systematic review of 364 patients from nine retrospective studies of women with stage I SEC, of whom 90% had stage IA disease, supports the use of adjuvant VBT, as it appears sufficient to guarantee high rates of local control without the need for pelvic RT [26]. Although there are no randomized trials for patients with non-invasive stage IA SEC comparing VBT with observation, it is common practice to offer this treatment to selected patients. The NCCN guidelines support the use of VBT with or without chemotherapy in these patients, as well as observation. Similarly, the ESGO/ESTRO/ESP guidelines consider VBT as an option to observation to decrease the rates of vaginal recurrence, especially in women aged 60 or above [6].

Patients with intermediate-risk disease in our cohort were treated with sequential chemotherapy and radiotherapy in the form of VBT (70%) or pelvic RT with or without VBT boost (20%), in alignment with current NCCN guidelines [14]. Notably, this cohort of patients was treated in an era where erring on overtreatment was the norm, prior to ESGO/ESTRO/ESP guidelines publications, explaining the absence of patients receiving VBT alone [6].

Regarding high-risk patients, a proportion of these were treated with single-modality treatments. Upon review of these cases, treatment decisions were guided chiefly by patient-related factors, including contraindications for RT such as extensive ulcerative colitis, complications that emerged during previous treatments such as fistula, advanced age, and comorbidities that made patients unlikely to tolerate combined modality treatment, amongst others. Other elements that might have influenced decision-making include the time span of treatment of this cohort (2002–2019), mainly before the publication of landmark trials like PORTEC-3 [13]. Therefore, the choice of adjuvant modality might have been based on retrospective data showing the benefits of chemotherapy in this disease. Despite this, the majority of patients with high-risk disease in our cohort (*n* = 19, 68%) received adjuvant treatment in the form of CRT in different sequences, the majority of which were CT and VBT, aligning with current recommendations [6].

In our study, we found a 3-year overall survival (OS) of 81% for the whole cohort, higher than the OS of 66% for stage I SEC with no adjuvant therapy reported by the 25th FIGO annual report in 2004 [27]. Even though the analyses were conducted at different times, the difference is interesting, considering that our cohort included patients with more advanced disease than the FIGO report. This could reflect a possible benefit from adjuvant therapy, especially because our 3-year DFS was 78%, similar to the 3-year OS of 81%.

CRT was associated with significantly improved OS and DFS compared to chemotherapy alone but not when compared to radiotherapy alone. This differs from the PORTEC-3 trial, which found a significant benefit in OS and failure-free survival (FFS) at 5 years for adjuvant concomitant CRT followed by four cycles of chemotherapy compared to EBRT alone in patients with high-risk EC [13,28]. Patients with SEC histology represented 16% (*n* = 105) of the study population, and in this subgroup analysis, significant benefits of CRT over RT in OS and FFS at 5 years were observed [24]. Moreover, molecular analysis of PORTEC-3 revealed that CRT provided a significant recurrence-free survival (RFS) benefit at 5 years for patients with stage I–III p53 mutated tumors, which represented 95% of the cases in which p53 status was known in our cohort [29]. Differences in results between our study and PORTEC-3 could be explained by the small size of the RT group (*n* = 6), which limits the statistical power for comparisons between groups, the heterogeneity of the RT technique with only 67% receiving pelvic RT in the RT alone group, the fact that most patients in the CRT group received VBT alone (54%), the sequential and not concomitant therapy in our CRT group, and differences in the number of cycles of chemotherapy, as most of our patients were planned for six cycles of carboplatin-paclitaxel given before radiotherapy.

GOG-258 compared the same CRT schedule of PORTEC-3 against six cycles of carboplatin-paclitaxel alone. This study primarily examined locally advanced EC but included stage I–II SEC with positive peritoneal washing cytology (*n* = 131, 18%). There were no significant differences in progression-free survival (PFS) between the treatment arms at 5 years (59% vs. 58%). This was consistent in the subgroup analysis for SEC, which only showed a tendency toward improved outcomes with CRT (HR 0.85, 95% CI 0.54–1.34) [28]. Even though this tendency was not statistically significant, it is aligned with our findings, in which patients treated with chemotherapy alone had significantly poorer OS and DFS than those in the CRT group. However, these results should be interpreted cautiously given the small number of patients in the CT group, the fact that we excluded patients with positive peritoneal washing, allowing only for patients with local disease, and inherent differences in the way CRT was delivered in our study [25,28]. 

As mentioned above, the outcomes of CRT, mostly comprised of patients receiving VBT and chemotherapy, are consistent with the results of GOG-249 [24]. This phase III trial assessed the combination of adjuvant VBT followed by three cycles of carboplatin-paclitaxel versus pelvic RT alone in patients with high-intermediate and early high-risk EC, including patients with stage I or II SEC (*n* = 88, 15%), and demonstrated no group OS or recurrence-free survival (RFS) differences. The EBRT group had significantly fewer nodal pelvic or para-aortic recurrences at 5 years (4% vs. 9%), but the rate of vaginal or distant recurrences did not differ significantly (approximately 2.5% and 18%, respectively). In subgroup analyses, VBT plus chemotherapy demonstrated a non-significant trend towards benefit regarding OS and RFS at 5 years when compared to pelvic RT alone (HR 0.77 [95% CI 0.35–1.67] and HR 0.81 [95% CI 0.44–1.52], respectively) in the SEC cohort [24]. Similarly, patients in our cohort showed no significant OS and DFS differences between the CRT and RT groups, but as cautioned previously, the small sample size of the RT group and the diversity of interventions in both treatment groups make it difficult to establish definitive conclusions.

Recurrent disease was seen in 29% of patients (*n* = 14), of which 79% (*n* = 11) had died at the time of analysis. After risk stratification, recurrence rates were 25% for intermediate-risk and 36% for high-risk patients, with distant metastases being a component of failure in the majority of cases in both groups. This highlights the aggressiveness of SEC despite early-stage diagnosis and multimodal adjuvant treatment.

Distant failure alone or in combination with local failure was the most common pattern of first recurrence. In GOG-249, the metastasis rate at 5 years was reported at 18% for the VBT plus chemotherapy group and RT alone group, while in PORTEC-3, the CRT group and RT alone group had 21.4% and 29.1% of distant failures, respectively, as a first recurrence [13,24]. In our cohort, the rates of metastases for the CRT and RT groups were 25% and 33%, respectively, both higher than the reports of the GOG-249 and PORTEC-3. This could reflect that our cohort was composed of a higher-risk population, as it exclusively included patients with SEC, which are known to have poorer outcomes than endometrioid histology [2,3,4]. Despite these findings being interesting, they remain hypothetical as the number of patients in each treatment subgroup in our cohort is too small to support any further analysis. 

Loco-regional failure (LRF) was 3% in the CRT group, similar to GOG-249 (2.5% of vaginal failure) and slightly higher than PORTEC-3 (1.4% of loco-regional failure as the first site of recurrence). This could reflect that most of our patients had a CRT schedule with VBT, similar to GOG-249, in contrast with PORTEC-3, which used concomitant CRT with pelvic EBRT [24,28]. Surprisingly, patients in the RT group of our cohort presented extremely high rates of loco-regional failure at 33%, but this reflects the outcomes of only two patients, one treated with VBT and presenting with pelvic and distant adenopathy, and another patient treated with pelvic EBRT, presenting with recurrence at the field margin and inguinal nodes. Interestingly, patients treated with CT alone presented with the same rate of local failure. However, the small subgroup sizes limit any definitive conclusions.

The toxicity analysis revealed that overall acute severe toxicity was present in 27% of the patients and was most commonly hematological (25%), mainly in patients receiving chemotherapy, with or without radiation. Only 2 patients presented with G3+ GI toxicity and 1 patient with G3+ GU toxicity. Overall, treatment compliance was limited by the completion of the chemotherapy (69%), as all patients in the CRT and RT groups completed their radiotherapy as planned. The 13 patients who did not complete all cycles of chemotherapy as initially planned did so for multiple acute reasons, often concurrent, ranging from presyncope or syncope (*n* = 4), hematologic toxicity (*n* = 3), myalgias (*n* = 3), severe neuropathy (*n* = 2), cardiotoxicity (*n* = 2), fatigue (*n* = 2), intolerance (*n* = 2), intractable nausea and vomiting (*n* = 1), enterocutaneous fistula post-surgery (*n* = 1), short-term memory loss (*n* = 1), and tongue thickening (*n* = 1). Despite the best supportive care (e.g., antiemetics, granulocyte-stimulating factor, wound care, etc.), chemotherapy delivery was altered from initial plans, which could affect disease outcomes and portend poorer survival. Limited sample sizes preclude subgroup analyses of treatment outcomes by treatment completion. Severe late toxicity was rare in our study, with two patients presenting hematological events (CRT group) and only one patient presenting with G3+ GU toxicity (CT group).

The main limitations of this study come from the retrospective nature of the design and the inherent selection bias associated, the heterogeneity of interventions with a variety of treatment approaches in each treatment group, including differences in chemotherapy cycles and drugs, RT modality and target volumes, treatment sequencing, and the small size of the CT and RT subgroups. However, our study included all eligible cases from our pathology database to reduce the risk of bias and, considering the rarity of SEC, emphasizes the poor prognosis of these patients, even with intermediate-risk disease, characterizes the patterns of failure, and highlights the need to further assess the roles of different adjuvant therapies in patients with stage I–II disease.

## 5. Conclusions

Stage I–II SEC has a high recurrence rate despite adjuvant treatments. CRT had better OS and DFS than CT alone and no significant differences compared to RT alone. Although limited by study design, small sample size, and treatment heterogeneity, our results are consistent with larger randomized trials showing similar survival outcomes between VBT plus CT versus EBRT alone. Distant failures alone or in combination with loco-regional recurrence were the most frequent patterns of failure, highlighting the need for novel systemic agents. Overall, the most frequent severe acute toxicity was hematological. Despite this, severe late toxicity was rare, even in the combined modality group.

## Figures and Tables

**Table 1:** Patient Characteristics.

	All Patients (*n* = 50)	CT (*n* = 6)	RT (*n* = 6)	CRT (*n* = 36)	Observation (*n* = 2)
Age at diagnosis, median (min–max)	69 (46–92)	70.5 (67–92)	65.5 (48–81)	66.5 (46–83)	89 (86–92)
BMI, median (min–max)	31.2 (20.6–48.9)	30.8 (21.5–33.8)	28.8 (23.9–48.9)	31.5 (20.6–47.6)	35.9 (32–39.8)
CEA at diagnosis (*n* = 35), median (min–max)	1.7 (0.4–6.5)	2.55 (1.4–3.6)	2.5 (1.6–3.6)	1.7 (0.4–6.5)	1.3
Ca-125 at diagnosis (*N* = 47), median (min–max)	14 (5–119)	13.5 (7–57)	14 (9–26)	14 (5–119)	19 (16–22)
ECOG Score 0, n	42	6	6	30	0
ECOG Score ≥1, n	8	0	0	6	2
Pelvic lymphadenectomy, n (%)	41 (82)	2 (33.3)	5 (83.3)	33 (92)	1 (50)
Para-aortic lymphadenectomy, n (%)	18 (36)	1 (16.7)	1 (16.7)	16 (44.4)	0
SNLD, n (%)	10 (20)	3 (50)	0	6 (17)	1 (50)
Follow-up (months), median	90.9	28.3	141.9	97.6	10
**Radiation Type**					
pEBRT, n (%), N = 42	10 (24)	N/A	3 (50)	7 (19)	N/A
VBT, n (%), N = 42	21 (50)	N/A	2 (33)	19 (53)	N/A
pEBRT + VBT, n (%), N = 42	11 (26)	N/A	1 (17)	10 (28)	N/A
**Tumor Characteristics**					
Figo 2009 Stage IA, n (%)	30 (60)	4 (67)	3 (50)	22 (61.1)	1 (50)
Figo 2009 Stage IB, n (%)	12 (24)	2 (33)	2 (33)	7 (19.4)	1 (50)
Figo 2009 Stage II, n (%)	8 (16)	0	1 (17)	7 (19.4)	0
ER+ (>5%), n (%), *N* = 41	32 (78) *	3/6 (50)	2/4 (50) *	26/29 (89.7) *	1/2 (50)
PR+ (>5%), n (%), *N* = 40	32 (80) *	3/6 (50)	3/4 (75) *	20/28 (71.4) *	0/2
P16+, n (%), *N* = 38	38 (100) *	6/6 (100)	2/2 (100) *	28/28 (100) *	2/2 (100)
P53+, n (%), *N* = 42	40 (95.2) *	6/6 (100)	4/4 (100) *	28/30 (93.3) *	2/2 (100)
LVI					
Negative, n	23	2	5	16	0
Focal, n	6	0	0	5	1
Extensive, n	21	4	1	15	1

*, missing data, percentages calculated with available data; BMI, body mass index; Ca-125, cancer antigen 125; CEA, carcinoembryonic antigen; ECOG, Eastern Cooperative Group; FIGO, International Federation of Gynecology and Obstetrics; LVI, lymphovascular invasion; N/A, not applicable; pEBRT, pelvic external beam radiotherapy; SLND, sentinel lymph node dissection; VBT, vaginal brachytherapy.

**Table 2:** Patterns of recurrence by treatment group.

First Site of Recurrence	CT (*n* = 6) N (%)	RT (*n* = 6) N (%)	CRT (*n* = 36) N (%)
Loco-regional	2 (33)	0	0
Distant	1 (17)	0	8 (22)
Loco-regional + distant	0	2 (33)	1 (3)

CRT, chemoradiotherapy; CT, chemotherapy; RT, radiotherapy.

**Table 3:** Characteristics of patients with disease recurrence.

FIGO 2009 Stage	LVI Status	Adjuvant Treatment	Site(s) of Recurrence	Time to First Recurrence (Months)	Salvage Therapy	Months Alive after Recurrence	Mortality Status
IA	Extensive	CRT	Distant (lung, para-aortic LN, inguinal LN, mediastinum)	82	CT	8.5	Dead
II	Extensive	CRT	Distant (field border para-aortic LN)	10	RT	49.4	Dead
II	Extensive	CRT	Distant (lung, non-para-aortic LN)	14	Observed	1.8	Dead
IB	Extensive	CT	Locoregional (vagina, LN)	10	Observed	12.7	Dead
IA	Focal	CRT	Distant (lung, brain)	39	SRS to brain only; lung observed	5.2	Dead
IB	None	CRT	Distant (liver)	20	Megestrol and tamoxifen	16.8	Dead
IA	None	RT	Locoregional (pelvic LN) Distant (peritoneal carcinomatosis)	14	CRT	15.1	Dead
IB	Extensive	CT	Locoregional (vagina)	12	Surgery, CT	20.6	Dead
IA	None	CRT	Locoregional (external iliac LN) Distant (inguinal LN)	46	RT	13	Dead
IA	Extensive	CT	Distant (liver, peritoneal carcinomatosis, para-aortic-, periportal-, hepatogastric LN)	12	CT	5.1	Dead
IA	None	RT	Loco-regional (out-of-field vagina, above-field common iliac LN) Distant (inguinal LN)	119	Surgery, CRT	129.4	Alive
IA	Extensive	CRT	Distant (lung)	18	RT	76.4	Alive
IA	Extensive	CRT	Distant (peritoneal carcinomatosis)	17	Chemo	7.3	Dead
II	Extensive	CRT	Distant (lung)	24	Chemo, RUBY trial (placebo vs. dostarlimab)	24	Alive

LVI, lymphovascular invasion; LN, lymph node; CT, chemotherapy; CRT, chemoradiotherapy; RT, radiotherapy; SRS, stereotactic radiosurgery.

**Table 4:** Number of acute and late toxicity events as per CTCAE v4.0 among patients receiving adjuvant treatments.

Toxicity	None * N	G1 N	G2 N	G3 N	G4 N	G5 N
**Acute**						
GU	41	7	0	0	1	0
GI	12	30	5	2	0	0
Hematological	10	12	15	7	5	0
**Late**						
GU	43	4	1	0	1	0
GI	41	6	2	0	0	0
Hematological	36	9	2	2	0	0

G, Grade; GI, Gastrointestinal; GU, Genitourinary; *, number of patients with no recorded toxicity.

## Data Availability

The original contributions presented in the study are included in the article and Supplementary Material; further inquiries can be directed to the corresponding authors.

## Supplementary Materials

The following are available online at https://www.mdpi.com/article/10.3390/curroncol31070277/s1, supplemental document for details of re-irradiation.
