# Supplementary material for: Adjuvant Treatment of Stage I–II Serous Endometrial Cancer: A Single Institution 20-Year Experience
Source: Curr Oncol. 2024 Jun 29;31(7):3758–70. doi: 10.3390/curroncol31070277 (PMC11276548; doi:10.3390/curroncol31070277)
Supplement: Supplementary file 1 [file curroncol-31-00277-s001.zip › curroncol-3044676-SI.pdf]

Supplement section 1:

There are 4 patients in question. Initial adjuvant treatment detail, as well as salvage therapies are as follows:

1. Patient in row 2 Adjuvant received CRT (RT type: EBRT + Brachy): upon recurrence at a para-aortic lymph node at the radiation field border, she received 54Gy/30. A higher dose of radiation was used as disease recurrence was at the field border of the previous radiation field. Radiotherapy was delivered safely with no adverse effects.
2. Patient in row 7 received Adjuvant RT (Brachy only): upon recurrence locoregionally at a pelvic lymph node and distant peritoneal carcinomatosis, she received 30Gy/10 and 5 cycles of carboplatin + paclitaxel. A mildly hypo-fractionated, palliative dose was combined with systemic chemotherapy due to peritoneal disease. Radiotherapy was delivered safely with no adverse effects.
3. Patient in row 9 received Adjuvant CRT (RT Type: Brachy only): upon locoregional recurrence at an external iliac lymph node, and distant recurrence at an inguinal lymph node, she received 25Gy/5 with Simultaneous Integrated Boost (SIB) to 30Gy/5 to macroscopic disease. Stereotactic body radiotherapy was used for local control at the nodes, as she previously only received brachytherapy and no previous external beam radiotherapy. Radiotherapy was delivered safely with no adverse effects.
4. Patient in row 11 received adjuvant RT (RT type: EBRT only): upon locoregional recurrence at the vagina (out of the previous radiation field) and common iliac lymph node, and a distant inguinal lymph node, she received surgery, then 45Gy/25 and 6 cycles of carboplatin + paclitaxel. Radiotherapy was delivered safely with no adverse effects.
